# Real-time airplane detection using multi-dimensional attention and feature fusion

**DOI:** 10.7717/peerj-cs.1331

**Published:** 2023-04-03

**Authors:** Li Li, Na Peng, Bingxue Li, Hao Liu

**Affiliations:** 1 School of Information and Electrical Engineering, Hebei University of Engineering, Handan, Hebei, China; 2 State Nuclear Power Demonstration Plant Co. Ltd, Weihai, Shandong, China

**Keywords:** Remote sense image, Airplane detection, Attention module, Feature fusion, Lightweight

## Abstract

The remote sensing image airplane object detection tasks remain a challenge such as missed detection and misdetection, and that is due to the low resolution occupied by airplane objects and large background noise. To address the problems above, we propose an AE-YOLO (Accurate and Efficient Yolov4-tiny) algorithm and thus obtain higher detection precision for airplane detection in remote sensing images. A multi-dimensional channel and spatial attention module is designed to filter out background noise information, and we also adopt a local cross-channel interaction strategy without dimensionality reduction so as to reduce the loss of local information caused by the scaling of the fully connected layer. The weighted two-way feature pyramid operation is used to fuse features and the correlation between different channels is learned to improve the utilization of features. A lightweight convolution module is exploited to reconstruct the network, which effectively reduce the parameters and computations while improving the accuracy of the detection model. Extensive experiments validate that the proposed algorithm is more lightweight and efficient for airplane detection. Moreover, experimental results on the airplane dataset show that the proposed algorithm meets real-time requirements, and its detection accuracy is 7.76% higher than the original algorithm.

## Introduction

With the rapid development of remote sensing technology and sensor technology, the quantity and quality of remote sensing images have been greatly improved in the last few decades, which can be used to describe various objects on the Earth’s surface, such as airports and buildings (Nie & Huang, 2021). Airplanes are one of the main objects of remote sensing images, and the accurate detection of airplanes plays an important role in military and civil fields. In the military field, it can quickly obtain intelligence and carry out strategic deployment by using of remote sensing images, and all-weather real-time accurate detection of airplane parked in military airports is carried out (MingJun et al., 2021). In the civil field, it can reduce the occurrence of airplane crashes and query the location of passenger airplane accurately and quickly by using of airplane object detection (Tang et al., 2022). Therefore, it is very important both in theory and practical to design a fast and accurate remote sensing image airplane object detection algorithm.

Object detection methods can be classified into two categories: artificial features-based methods (Shanmuganathan, 2016) and deep learning-based methods (Xu et al., 2021; Chen et al., 2020; Li, Li & Zhou, 2022; Zhang & Zhou, 2022). Artificial features-based methods such as scale-invariant features (Chen et al., 2020), directional gradient histograms (Xiao et al., 2020), which are all manually designed for detection, the transformation capability is weak, time-consuming and labor-intensive. Also, it is difficult to meet the requirements of real-time and high accuracy. Deep learning-based methods have been widely used in recent years due to its good independent learning ability and generalization ability. It can be divided into two categories methods (Liu et al., 2020): one is two-stage object recognition based on regional recommendation such as R-CNN and the improved methods (Wang, Shrivastava & Gupta, 2017; Girshick et al., 2014; Ren et al., 2015; Dollár & Girshick, 2017), *etc*. These methods generate object candidate frames first and then perform classification and regression on the images in the candidate frames. Two-stage object recognition methods have high positioning accuracy. But the detection speed of those works is slow and they still perform poorly for the real-time requirements of airplane detection. The other is regression-based single-stage object recognition, such as Single Shot Detector (SSD) (Liu et al., 2016; Zhang et al., 2020; Yang & Wang, 2019), You Only Look Once (YOLO) series (Redmon et al., 2016; Bochkovskiy, Wang & Liao, 2020; Redmon & Farhadi, 2017, 2018), *etc*., which use end-to-end methods to classify and regress the object image directly. Regression-based single-stage object recognition methods have a fast detection speed, which can meet the real-time requirements. But there are still certain defects in accuracy, especially for small objects such as airplanes in remote sensing images, which are more susceptible to factors such as noise, light intensity, and weather. Also, those algorithm generally have a low recall rate, missing detection and misdetection, *etc*. A large number of scholars at home and abroad have made many explorations and improvements to solve the above problems. Zhang et al. (2021) modified the depth of the cross stage partial (CSP) module in Yolov4-tiny to strengthen feature extraction, at the same time, it built a spatial pyramid pooling (He et al., 2015) module to increase the receptive field of the feature map and improve the recall rate. However, the improvement of network accuracy through the superposition of structures has undoubtedly increased a large number of parameters, and the detection speed was greatly reduced. Zhou, Xu & Zhu (2021) introduced the DeBlurring (Pan et al., 2018) algorithm and the dark channel prior algorithm (He, Sun & Tang, 2010) to increase the network’s adaptability to the weather environment, while adding a detection layer to improve the detection ability of small objects in rain and fog. However, the image processing also increased time for network training. Hou, Ma & Zang (2021) added an improved anti-residual block to the feature fusion network, which it make feature mapping operate in high dimensions and improve the ability of the model to detect small objects. However, the initial feature extraction layer mechanism has not changed and the shallow feature information cannot be fully utilized, and there is still a problem of missed detection on very small objects.

From the perspective of detection accuracy and speed, this article proposes an accurate and efficient airplane detection algorithm (AE-YOLO). The main contributions of this work are summarized as follows:

1. Using a lightweight multi-dimensional attention mechanism in the backbone network to optimize the feature expression ability of the backbone network and enhance the network’s learning ability for small samples.
2. Using the weighted two-way feature pyramid operation fuses features to obtain richer semantic information and location information.
3. The recognition network was reconstructed by exploiting a lightweight module, which greatly improves detection accuracy and speed of airplane objects.
4. We experiment on the airplane dataset, and the results show that the algorithm in this article has a better detection performance than Yolov4-tiny in the detection of airplane images with ground looks similar to background in color, densely distributed, and overexposed.

The rest of this article is organized as follows: an airplane object detection algorithm named AE-YOLO is proposed in “AE-YOLO”. “Experimental process and result analysis” presents the experimental environment, datasets, evaluation metrics used in the experiments and the performance of each improved module. Finally, a concise conclusion of the whole work is given in “Conclusion”.

## Ae-yolo

### Network structure

**Figure 1:**
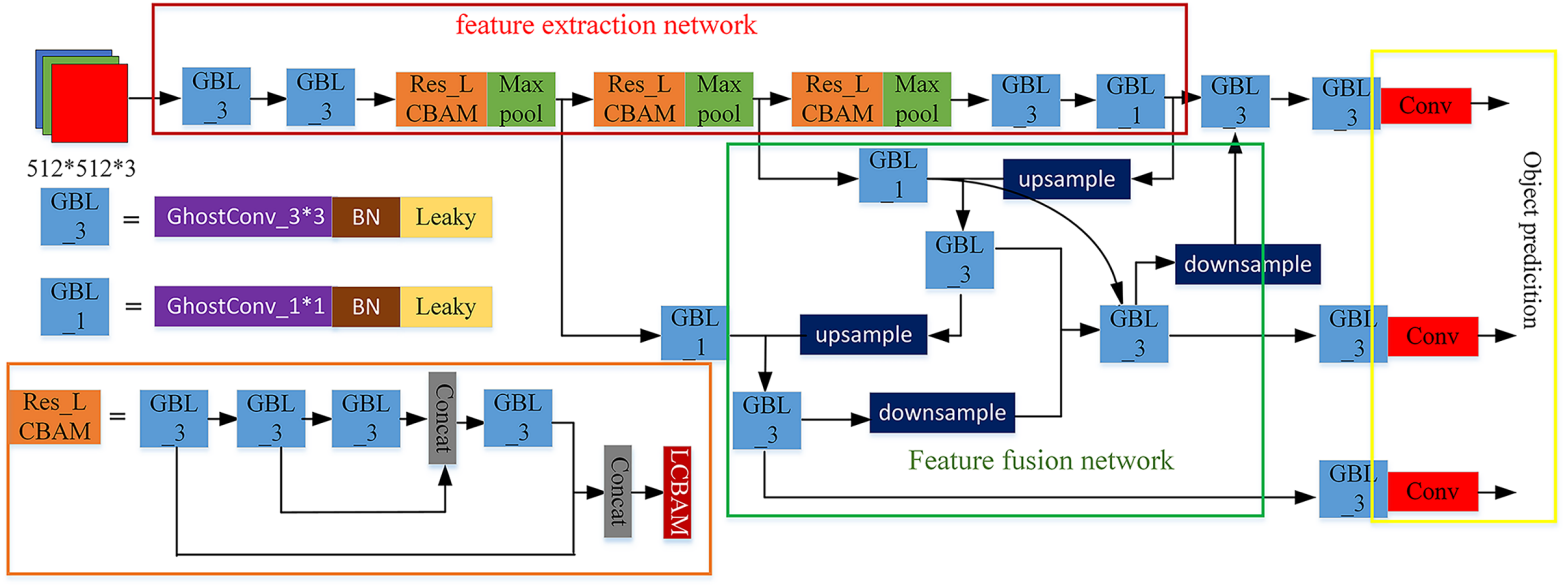
AE-YOLO structure.

This section will introduce the proposed network model AE-YOLO in detail. Its structure is shown in Fig. 1. It mainly consists of three parts: the lightweight multi-dimensional attention backbone feature extraction network (ghost CSP) (shown in the red box of Fig. 1), the weighted feature fusion network BiFPN (Tan, Pang & Le, 2020) (shown in the green box of Fig. 1) and the YOLO-head detection (shown in the yellow box of Fig. 1). Taking the input image $512 \times 512$ as an example, the detection process is mainly divided into three steps:

**(a) Feature extraction.** First, the features are extracted and down-sampled through two ghost (Han et al., 2020) convolution operations. At the same time, BatchNormalization (Jian-Wei et al., 2020) normalization and LeakyRelu (Schmidt-Hieber, 2020) activation functions are added after the convolution operation to avoid gradient disappearance and gradient descent problem, and it can improve the generalization of the network. Then the obtained feature map goes through the ResBlock_LCBAM module three times. ResBlock_LCBAM divide the feature map of the base layer into two parts by using the CSP structure, and it adds a residual edge next to the residual block through cross-layer connection. It then merges them and extracts important channels and spatial information through the LCBAM attention module, which reduces the missed detection rate of small objects. Finally uses maxpooling for down-sampling.

**(b) Feature fusion.** The three-layer feature maps with resolutions of $64 \times 64$, $32 \times 32$, and $16 \times 16$ were selected from the backbone network, and the two-way weighted feature pyramid was used for feature fusion. By assigning a weight to each branch, the weight information of each branch was learned through end-to-end network training and therefore improves the utilization of features.

**(c) Object prediction.** The input image is divided into $s \times s$ grids. Each grid predicts three bounding boxes of different sizes and the coordinate offset value and confidence level corresponding to each bounding box. The predicted offset value was decoded through the decoding method, and it can generate the final bounding box coordinates. Finally, by setting a threshold, the non-maximum suppression algorithm (Salscheider, 2021) is used for post-processing, and the result with the highest score is selected and output it.

### Multi-dimensional attention feature extraction network

In the convolutional neural network, the airplane object is susceptible to interference and is not conducive to the detection of small objects in the complex background because the airplane object in the feature map has the same importance as other complex backgrounds. In order to increase the recognition ability of feature maps for specific areas and specific channels, AE-YOLO introduces a multi-dimensional attention module to increase the importance of the object area and improve the detection ability.

**Figure 2:**
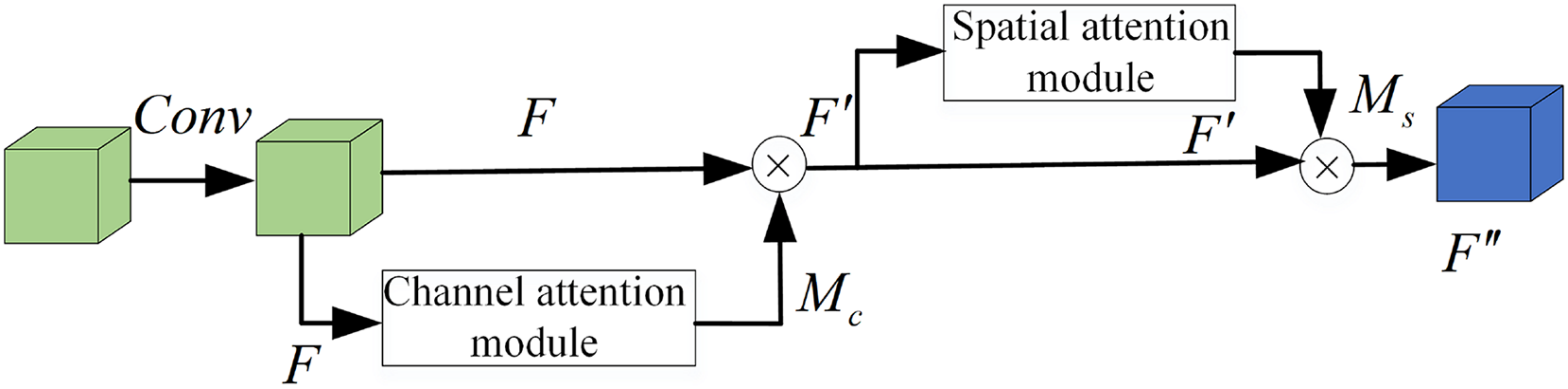
LCBAM module.

We propose a plug-and-play lightweight multi-dimensional attention module LCBAM (Light-CBAM) based on the attention mechanism of CBAM (Woo et al., 2018) and ECA (Wang et al., 2020). By using channel attention and spatial attention, it makes the feature map to infer the channel attention feature map with each channel as the unit and the spatial attention feature map with the pixel point as the unit in the order of two independent dimensions, and then multiplies the generated attention feature map by the input feature map for adaptive feature refinement. At the same time, one-dimensional convolution is used in the channel part to carry out cross-channel interactive fusion of one-dimensional channel attention, which can avoid the loss of information caused by fully connected layer compression and increases the parameter of the model to a constant level. This allows the network to better focus on the questions of “what” and “where” the object is. Figure 2 shows the overall flow of the LCBAM module.

Given an intermediate feature map *F*, one-dimensional channel attention ${M_c}$ is generated through the channel attention module first. Then multiply the channel attention ${M_c}$ by the input feature map with element to obtain the feature map *F′* and input it into the spatial attention module to generate space attention module ${M_s}$, and then multiply the spatial attention by *F′* to generate the final feature map *F′′*. The expressions can be defined as follows:

(1) $$F^{\prime} = {M_c}(F) \otimes F$$

(2) $$F^{\prime\prime} = {M_s}(F^{\prime}) \otimes F^{\prime}$$

**Figure 3:**
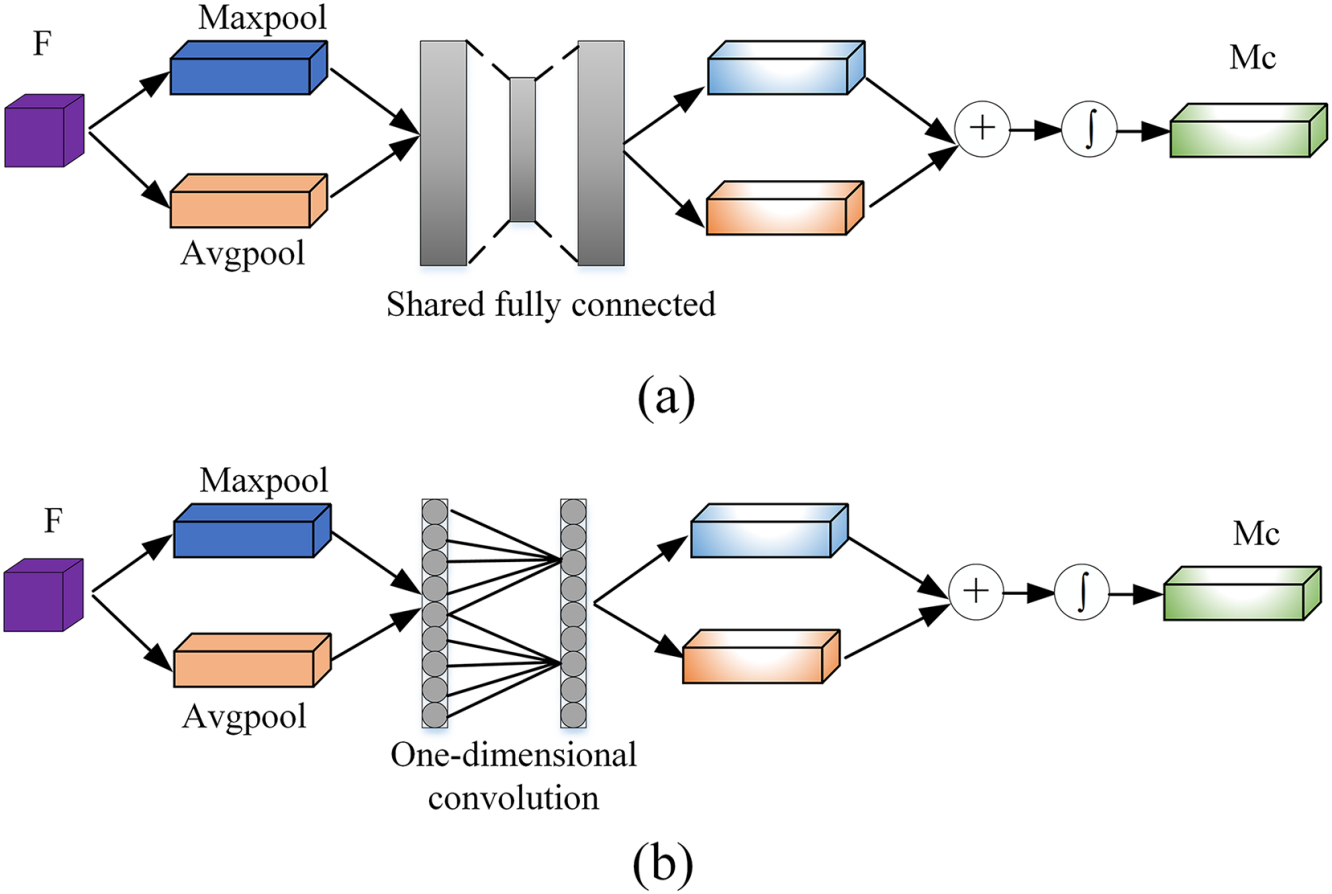
The pipeline of channel attention: (A) original channel attention module and (B) the proposed channel attention module.

To generate the channel attention module, follows these steps. First, using the global average pooling and maxpooling operations on the feature map *F* to increase the receptive field of the feature map and to aggregate the global spatial information of the feature map. Two different feature descriptors $F_{avg}^c$ and $F_{max}^c$ are generated at the same time. Then using a one-dimensional convolution with a convolution kernel length of $k$ to perform cross-channel fusion of $k$ channels in the neighborhood of the channel, and finally add the two features after the convolution by element and use the sigmoid (Mourgias-Alexandris et al., 2019) activation function to generate a one-dimensional channel attention ${M_c}$. Figure 3 shows a comparison diagram of CBAM channel attention and our proposed channel attention. The initial fully connected layer is modified to reduce the information loss caused by compression of the fully connected layer.

${M_c}$ can be defined as Eq. (3):

(3) $${M_c}(F) = \sigma (f_1^k(Avgpool(F)) + f_1^k(Maxpool(F))) = \sigma (f_1^k(F_{avg}^c) + f_1^k(F_{max}^c))$$

where $f_1^k$ represents the one-dimensional convolution operation with the convolution kernel size $k$. $\sigma ( \cdot )$ denotes Sigmoid function. The size of $k$ is defined as Eq. (4) (Wang et al., 2020).

(4) $$k = |{{log(C) + 1} \over 2}{|_{odd}}$$

where *C* represents the number of channels of the input feature map, and $| \cdot |$ represents the nearest odd number. The generation steps of the spatial attention module can be described as follows: average pooling and maxpooling of *F′* can be performed in the spatial dimensions respectively. $F_{avg}^s$ and $F_{max}^s$ descriptors in two spatial dimensions are generated, and then the two spatial features are spliced together to form a new spatial feature map, which its spatial channel number is 2. Finally, convolution is performed by convolution operation and the sigmoid activation function is used to generate a two-dimensional spatial attention mechanism ${M_s}$. This operation can be defined as Eq. (5):

(5) $${M_s}(F^{\prime}) = \sigma ({f^{7 \times 7}}([AvgPool(F^{\prime});MaxPool(F^{\prime})]))$$

where ${f^{7 \times 7}}$ denotes a $7 \times 7$ convolution operation, $\sigma$ indicates the use of the sigmoid activation function.

**Figure 4:**
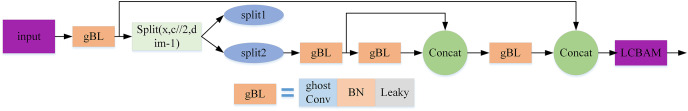
Resblock_LCBAM module.

Since the initial backbone network consists of three standard convolutions and three ResBlocks, each ResBlock module only performs four convolution operations and the entire backbone network uses only 15 convolutions to extract the input image, the network structure is too simple. When the airplane object is very small or the background color is similar, the extracted airplane object information is less and the network’s ability to distinguish useful features is weak, which is easy to cause missed detection. In order to solve this problem, AE-YOLO is based on the idea that shallow feature structure is simple and universal and the deep features are more complex and abstract (Fu et al., 2021), an improved attention mechanism LCBAM (Resblock_LCBAM module) is added after the convolution operation in the three deeper Resblock modules. The structure of Resblock_LCBAM module is shown in Fig. 4. The attention mechanism is applied to the feature map generated by the convolution operation so that the network self-learns the importance of each channel and spatial information and optimizes the weight information in the feature map, and the channel and space area are used that makes the substantial contribution. It means that the airplane object can be effectively distinguished from the background information without causing the parameters to increase in numbers, provides a good foundation for the subsequent feature fusion and prediction.

### Weighted two-way feature fusion network

**Figure 5:**
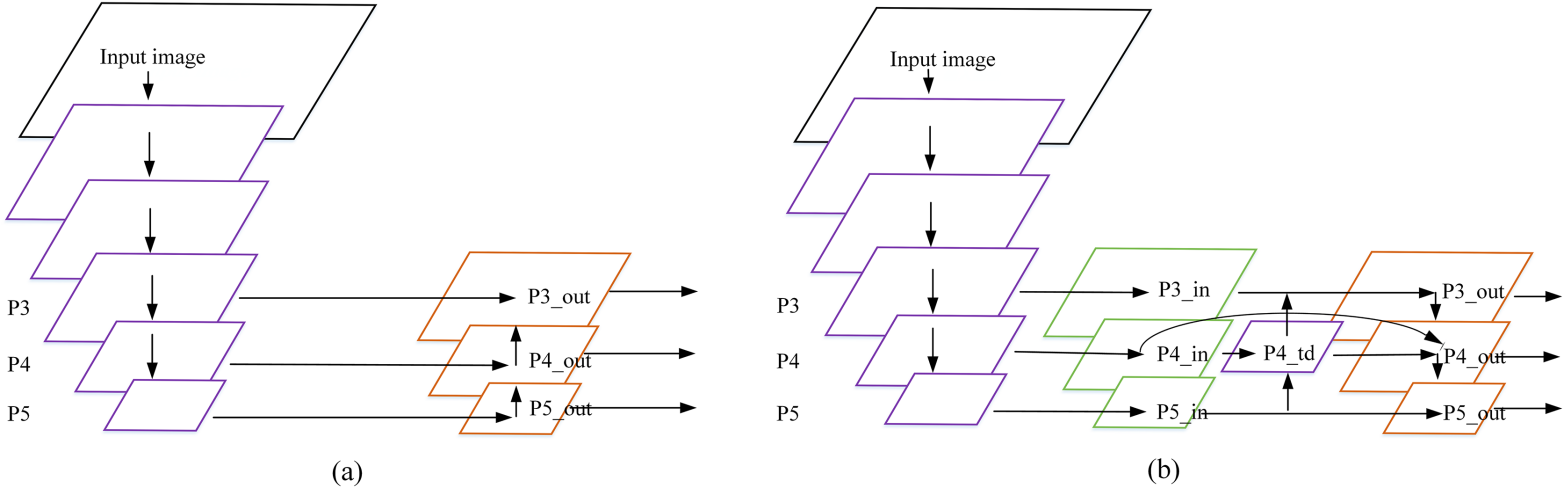
Feature fusion network: (A) FPN and (B) BiFPN.

The initial Yolov4-tiny uses FPN (Feature Pyramid Networks) (Kirillov et al., 2019) as the feature fusion network, and the P4 and P5 feature maps are obtained by using the backbone network to compress with 16 times and 32 times respectively. The channel is spliced with upsampling P5 and P4, two feature maps with sizes of $26 \times 26$ and $13 \times 13$ are generated and input them into the detection layer for detection. This means that many details and position information of the object are lost in the feature map for objects with a size smaller than $16 \times 16$ pixels, which cannot meet the detection requirements for small objects. In the remote sensing image airplane dataset, small objects are in the majority and the pixel ratio is very small (Shi et al., 2021). Local features and detailed information will be lost by using deep feature maps. Therefore, the AE-YOLO model adds a layer of feature scale to improve the detection accuracy of small objects. The specific method is to perform upsampling by using the fusion of P5 and P4, and merge it with the feature map of P3 (compressed eight times) to generate a three-layer prediction layer. This is shown in Fig. 5A.

The fact that the high resolution of shallow features have richer detail information, the low resolution of deep features have richer semantic information. However, FPN simply performs a feature fusion with P4 and upsampling of the P5 feature layer, and the utilization of the feature information extracted from the backbone network is too low. In order to fully integrate the shallow detail information with the deep semantic information, the AE-YOLO network architecture adds a top-down channel fusion on the basis of FPN. In the meantime, by reference to the bidirectional feature fusion (BiFPN) (Tan, Pang & Le, 2020) idea, this article removes the small contribution nodes with only one input edge, and uses the residual method (He et al., 2016) to add one jump connection when the input and output nodes are in the same layer, so that more features are merged without increasing computational overhead. Finally, considering that the contributions of these different input features to the output features are not equal, learnable weights are introduced to learn the importance of different input features. Fast normalized fusion is used in the selection of weights. Under the premise of similar to softmax (Sharma, Sharma & Athaiya, 2020) in learning behavior and accuracy, the running speed on the GPU is increased by $30\%$. The definition is given by Eq. (6):

(6) $$O = \sum\limits_i {{{{w_i}} \over {\epsilon + \sum\nolimits_j {{w_j}} }}} \cdot {I_i}$$

where $i$ and $j$ represent the number of input feature maps by the feature fusion node, ${I_i}$ represents the input feature map matrix. $\epsilon = 0.0001$, ${w_i}$ and ${w_j}$ represent the weight of the input feature map. The final fusion method is shown in Fig. 5B, take the $P4\_td$ node for example, the expression is shown in Eq. (7):

(7) $$P4\_td = Conv({{{w_1} \cdot P4\_in + {w_2} \cdot resize(P5\_in)} \over {{w_1} + {w_2} + \varepsilon }})$$

where $P4\_td$ represents the first fusion result, which is composed of two branches. The two branches are the feature map obtained through $P5\_in$ upsampling and $P4\_td$, respectively. $resize( \cdot )$ represents the upsampling operation of feature map with the unified size. ${w_1}$ represents the weight occupied by $P4\_in$, and ${w_2}$ represents the weight occupied by $P5\_in$.

### Lightweight recognition network

In order to balance the detection accuracy and speed of the model, we took the perspective of lightweight, the entire network is reconstructed by the lightweight ghost module, which reduces a lot of redundancy in the intermediate feature maps of mainstream convolutional neural networks, and greatly reduces the computation and parameters while maintaining the accuracy.

**Figure 6:**
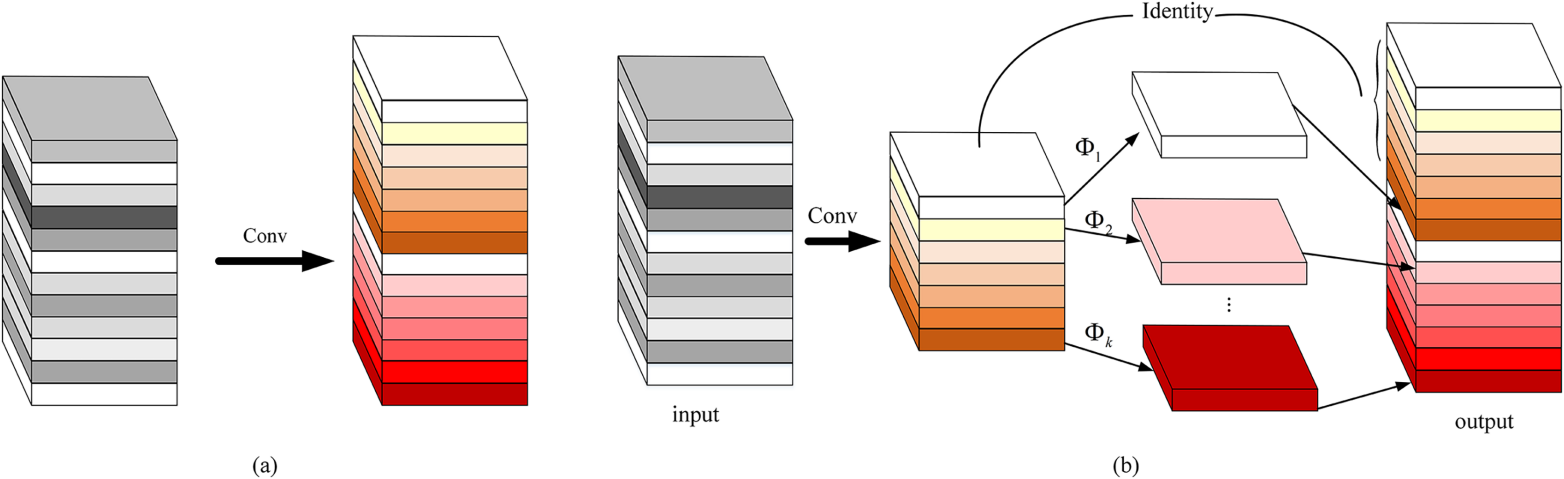
Comparison of convolution methods: (A) standard convolution and (B) ghost convolution.

Given an input feature map $X \in {R^{c \times h \times w}}$, where $c$ is the number of input channels, $h$ and $w$ are the length and width of the input feature map, a convolution kernel $f \in {R^{c \times k \times k \times n}}$ is used, $k$ is the size of the convolution kernel, and $n$ is the number of convolution kernels, then using standard convolution to generate the feature map as shown in Fig. 6A. The expressions can be defined as follows:

(8) $$Y = X * f + b$$

where $*$ represents the convolution operation, $b$ represents the bias term. $Y \in R^{h^\prime \times w^{\prime} \times n}$ represents the output feature map with the number of output channels $n$, and the height and width are $h^{\prime}$ and $w^{\prime}$ respectively, then the computation amount of the standard convolution is $n \times h^{\prime} \times w^{\prime} \times c \times k \times k$ (Albawi, Mohammed & Al-Zawi, 2017).

However, the ghost module uses the cheap linear operations to complete the generation of feature redundancy, which reduce a large number of convolution operations, specifically as follows: First, the standard convolution is used to generate the inherent features $Y^{\prime} \in R^{h^{\prime} \times w^{\prime} \times m}(m \le n)$ of the m-layer channel, and then based on *Y′*, a simple linear $m$ change is used to obtain the ghost feature. Finally, the two sets of feature maps are spliced in the specified dimension. As shown in Fig. 6B, the expressions are as shown in Eqs. (9) and (10):

(9) $$Y^{\prime} = X * f^{\prime} + b$$

(10) $${y_{ij}} = {\Phi _{i,j}}(y^{\prime}{_i}),\forall i = 1, \cdots ,m,j = 1, \cdots ,s$$

where Eq. (9) represents that $m$ intrinsic features $(m \le n)$ are generated by using standard convolution. $y^{\prime}_i$ represents the i-th inherent feature in *Y′*. ${\Phi _{i,j}}$ represents the j-th linear operation which is used to generate the j-th ghost feature ${y_{i,j}}$, that is to say, $y^{\prime}_i$ has one or more ghost features $\{ {y_{ij}}\} _{j = 1}^s$. Finally, $m \times s = n$ feature maps are generated as the output of the ghost convolution module, so using the ghost module can generate the same number of feature maps as normal convolution.

In order to improve the processing speed, $3 \times 3$ or $5 \times 5$ linear operations are used. Suppose the average size of the convolution kernel used in the linear operation part is $d \times d$, then the speedup ratio of the standard convolution and the ghost module convolution can be defined as Eq. (11):

(11) $$SR = {{{nh^{\prime}w^{\prime}ckk }\over{n{s^{ - 1}}h^{\prime}w^{\prime}ckk + (s - 1)n{s^{ - 1}}h^{\prime}w^{\prime}dd} }} = {{ckk}\over {{s^{ - 1}}ckk + (s - 1){s^{ - 1}}dd}} ={{sc}\over{s + c - 1}} \approx s$$

where the size of $k$ and $d$ is the same, and $s \ll c$, so the computation amount of the standard convolution obtained by the final simplification is s times that of the ghost module. This article draws on the idea of ghost module and replaces all standard convolutions in the entire object recognition network with ghost convolutions, which significantly improves the detection speed of the algorithm.

## Experimental process and result analysis

### Experimental environment

The computer hardware and software configuration used in this experiment are as follows. CPU is Intel(R) 3.3Core(TM) i7-10750H and GPU is Nvidia GeForce GTX 1660Ti. The experiments are implemented on the PyTorch deep learning framework with 16 GB RAM in Windows computer. Pycharm compilation software and python programming language is used.

### Dataset

At present, there is a lack of public datasets suitable for deep learning training in the research of remote sensing image airplane object detection. The airplane datasets used in this article are derived from the remote sensing image datasets RSOD (Long et al., 2017) and UCAS-AOD (Zhu et al., 2015), which are photographed and marked by Wuhan University and University of Chinese Academy of Sciences respectively. These mainly contain 1,446 images and 12,475 airplanes with dense distribution, ground looks similar to background in color, overexposure, normal distribution, *etc*. For the flying aircraft in the remote sensing image, because the distance between the shooting position and the aircraft is much larger than the distance between the aircraft and the ground, it can not be seen that the aircraft is flying, so the flying aircraft can be approximately regarded as taking the ground as the carrier.

### Evaluation metrics

In order to compare detection performance of the algorithm before and after improvement on objects such as blur and exposure in the airplane dataset, this article selects the precision rate (*P*, precision), the recall rate (*R*, Recall), and the average precision (*AP*, Average Precision) to evaluate the detection efficiency of the algorithm. Detecting each image required time ( $t$) represents the detection rate of the model. *P*, *R* and *AP* are defined as follows:

(12) $$P = {{TP} \over {TP + FP}}$$

(13) $$R = {{TP} \over {TP + FN}}$$

(14) $$AP = \int_0^1 P (R)dR$$

where *TP* represents the number of true detected positive samples, *FP* represents the number of false detected positive samples, and *FN* represents the number of false detected negative samples. *R* indicates that all true objects are detected by the algorithm, and *P* indicates that how many of the detected objects are true objects. *AP* represents the average detection rate of a single category.

### Analysis of results

#### Training process

**Figure 7:**
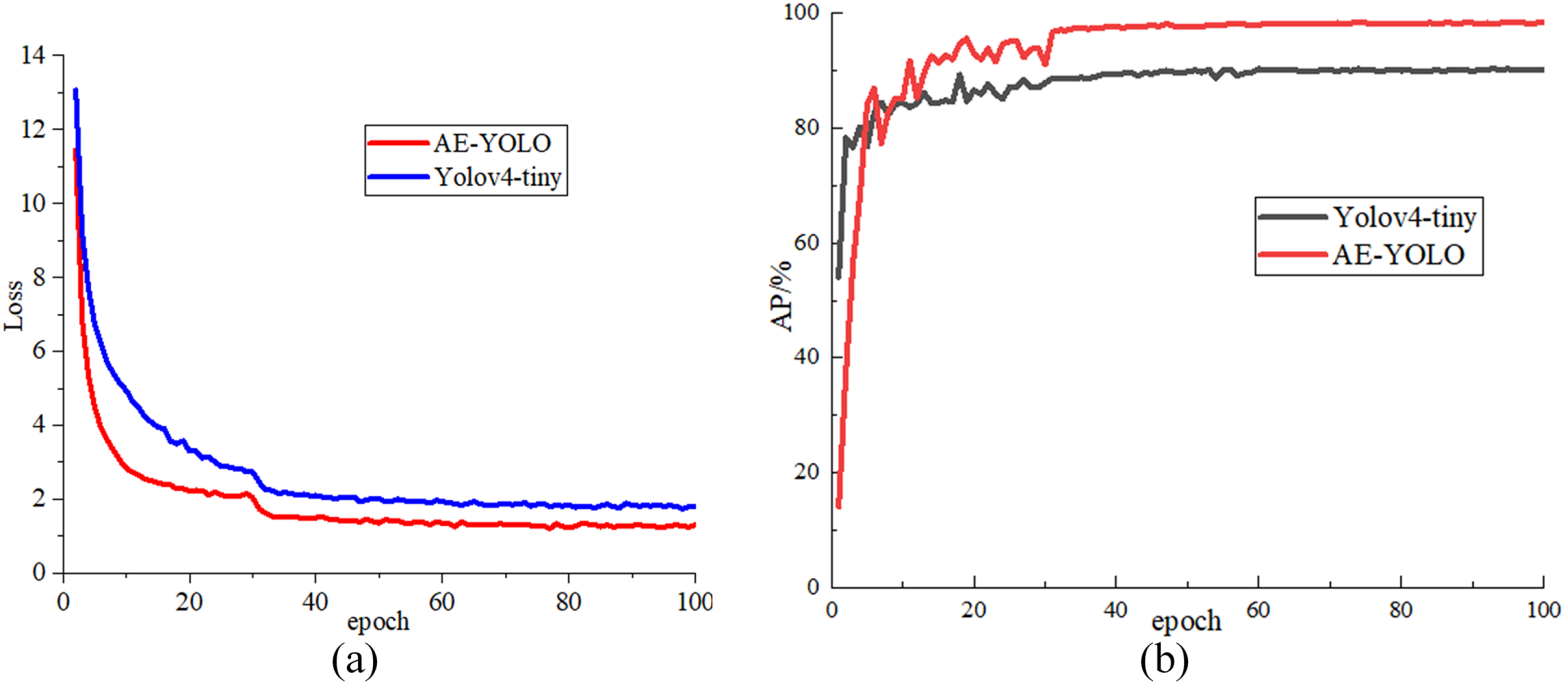
Comparison of loss value and AP value during training: (A) loss drop curve comparison and (B) AP rise curve.

In the experiment, the initial learning rate is set to 0.001. The network is trained for a total of 100 epochs, and the learning rate is changed to 0.1 times that of the previous one every 30 epochs. The batch_size is set to 4, and the adam optimizer is used to optimize the network. The input image size is $512 \times 512$ for training all models. In the training process, the mosaic data enhancement method is used to process the data set. At first, four images are randomly selected and cropped and all splices are combined together are combined together training data, which improves the diversity of the dataset. Then, it is randomly assigned in a ratio of 8:2, 80% is used as training data, 20% is used as test data, and 90% of the training set is used for training and 10% for validation. Figure 7 shows the loss drop curve and the AP rise curve of the initial Yolov4-tiny algorithm and the AE-YOLO algorithm, respectively. Along with the increasing of iteration times, both AE-YOLO and Yolov4-tiny gradually tend to be stable, and the final loss value converges to about 1.2 and 1.8 respectively. The AP of yolov4-tiny is stable at around 90% and the AP value of AE-YOLO is stable at around 98%. The detection effect of AE-YOLO is better, and it is more suitable for the detection of small airplane objects.

#### Validity analysis of the proposed method

(a) Performance of different attention mechanisms

**Figure 8:**
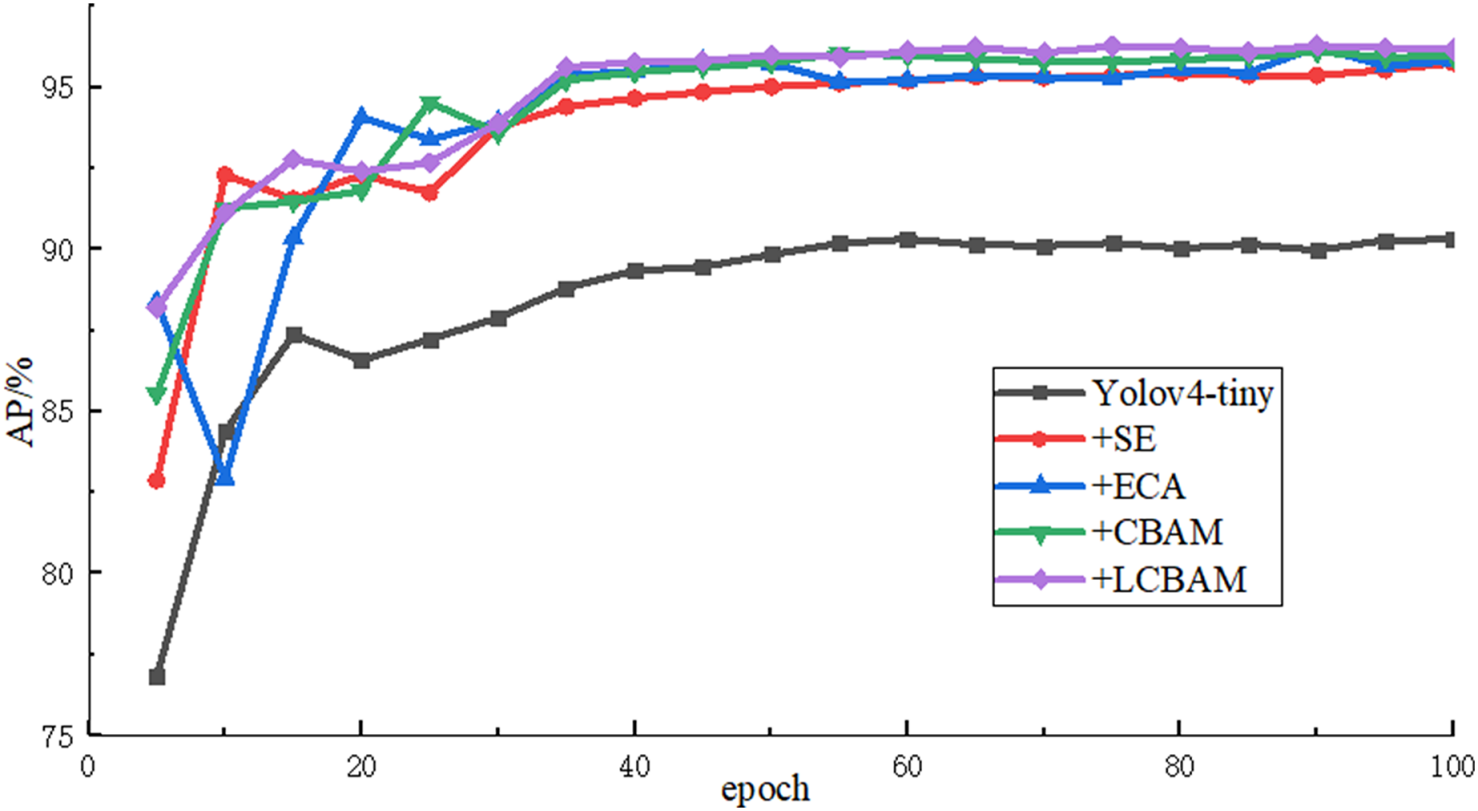
AP rise curves of different attention mechanisms.

In order to verify the effectiveness of the proposed attention mechanism, AE-YOLO uses SE attention mechanism, ECA attention mechanism, CBAM attention mechanism and improved LCBAM in the Yolov4-tiny initial backbone network for comparative experiments, and the comparison results are shown in Table 1. The results show that the AP and recall rate are greatly improved compared with the initial Yolov4-tiny regardless of which attention mechanism is used, and AP of the four models is improved by 5.45%, 5.53%, 5.76% and 5.89% respectively. It indicates that the attention mechanism can well optimize the feature map in the backbone network, which it can enhance the attention to important information, reduce the missed detection and improve the recall rate of small airplane objects through adaptive weight distribution. The LCBAM proposed in this article has the best detection effect. SE and ECA only use channel attention and ignore the spatial information of small objects in the feature map, and the accuracy improvement is less than CBAM. But ECA uses a local cross-channel interaction strategy without dimensionality reduction, aggregates the k neighborhoods information of the channel, and adaptively selects the size of the one-dimensional convolution kernel, which avoids the loss of information caused by the compression of the fully connected layer in SE, and its performance is 0.08% higher than SE and the number of parameter only increase 15. Although CBAM adds spatial attention and its detection accuracy is much improved compared with SE and ECA, the number of model parameters increases a lot and there is also the problem of information loss caused by the aggregation of fully connected layers. The LCBAM proposed in this article achieves the best final recall rate and detection accuracy by combining the advantages of CBAM and ECA. Compared with Yolov4-tiny, the recall rate and detection accuracy of the network with LCBAM increased by 8.41% and 5.89% respectively and the number of parameters only increased by 309. The detection time of each image is also only 1.48 ms slower. Figure 8 shows the rising curve of the detection accuracy of these models during the training process. As the number of iterations continues to increase, the AP values of these models continue to rise, and gradually become stable after epoch50. The results show that LCBAM has the highest detection accuracy and the best effect.

**Table 1:** Performance comparison of different attention mechanisms. The best and the second results are highlighted in bold and underline, respectively.

Model	R (%)	P (%)	AP (%)	Parameter increment $\downarrow$	t (ms) $\downarrow$
Yolov4-tiny	$85.45$	${\bf{96}}.{\bf{96}}$	$90.30$	${\bf{0}}$	${\bf{9}}.{\bf{05}}$
+SE	$92.74$	$94.54$	$95.75$	$43,\!008$	$10.75$
+ECA	$93.00$	$94.59$	$95.83$	15	10.29
+CBAM	93.56	$95.12$	96.06	$690,\!217$	$14.68$
+LCBAM	${\bf{93}}.{\bf{86}}$	95.14	${\bf{96}}.{\bf{19}}$	$309$	$10.53$

(b) Detection effect of different feature fusion networks

**Figure 9:**
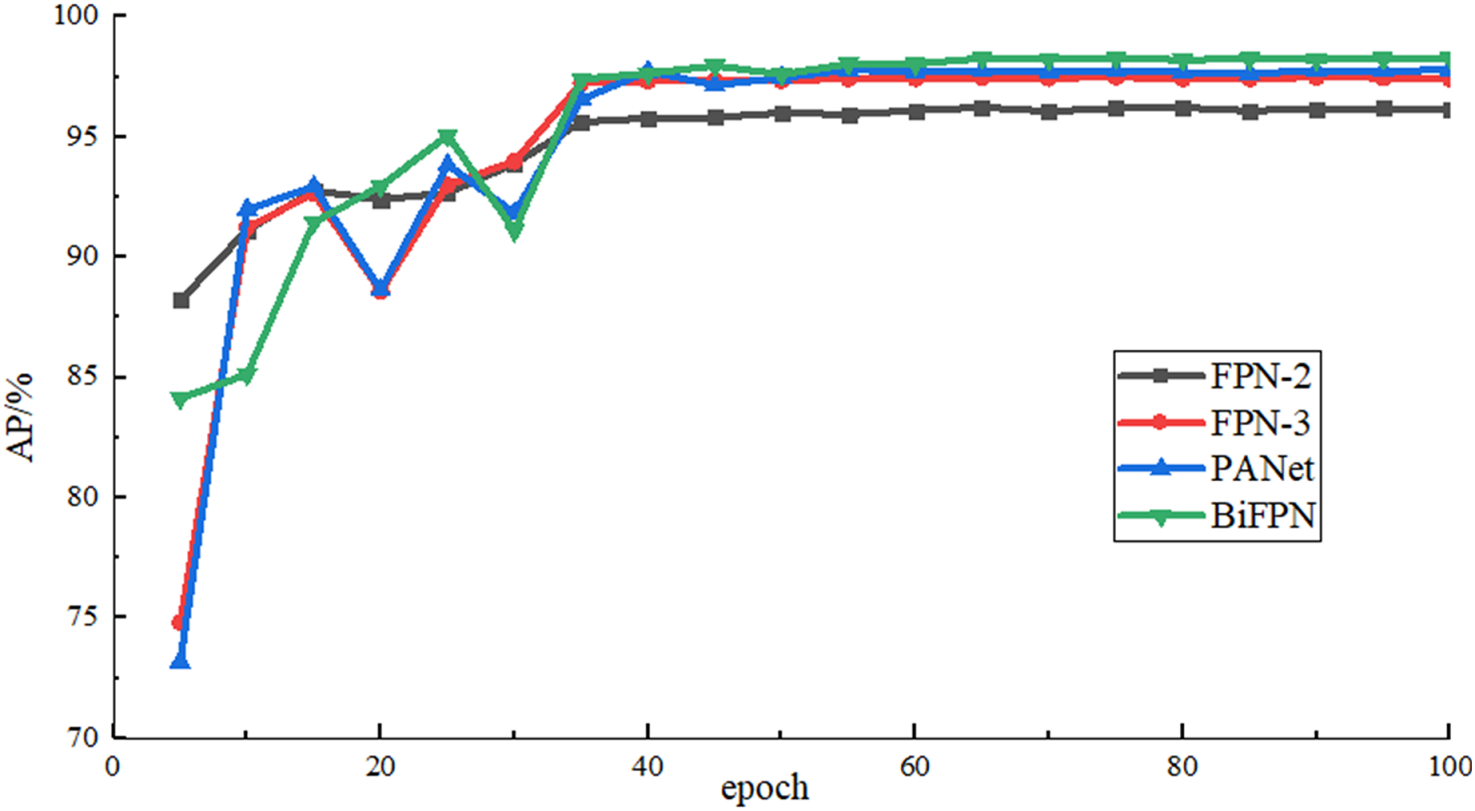
AP rise curve of different feature fusion networks.

In order to verify the effect of the bidirectional feature fusion network used in this article, the multi-scale feature fusion networks such as two-layer FPN, three-layer FPN, PANet and BiFPN are used for comparison in the case of using the same backbone feature extraction network (namely, adding LCBAM to the backbone network) and parameter settings, and the results are shown in Table 2. It can be seen that the two-layer FPN used in the initial Yolov4-tiny is not suitable for airplane data sets with many small objects and the detection accuracy is low. After adding a shallow feature map, it contains more detailed features. The detection accuracy is improved by 1.27% compared with two-layer, but there is only one-way feature fusion and the shallow feature information is not fully utilized. The top-down bidirectional feature fusion is added to PANet, which improves the algorithm’s ability to extract low-level features and improves the ability to locate small airplane objects. The detection accuracy is improved by 1.58% compared with the two-layer FPN. At the same time, in PANet, five consecutive convolutions are used to process the feature map after splicing, which improves the accuracy and also increases greatly the number of parameters. The BiFPN used in this article adopts bidirectional feature fusion similar to the PANet structure and assigns a weight to each input branch, which enables the network to learn the importance of each input branch autonomously, and achieves the highest detection accuracy among multiple scale feature fusion methods. Compared with the original FPN-2, the detection time is increased by 4.59 ms. Figure 9 shows the rise curve of AP value during training by using different feature fusion networks.

**Table 2:** Performance comparison of different feature fusion networks. The best and the second results are highlighted in bold and underline, respectively.

Model	R (%)	P (%)	AP (%)	Parameter increment $\downarrow$	t (ms) $\downarrow$
FPN-2	$93.86$	$95.14$	$96.19$	${\bf{0}}$	${\bf{10}}.{\bf{53}}$
FPN-3	${\bf{94}}.{\bf{55}}$	$96.64$	$97.46$	445,330	13.31
PANet	$94.21$	97.41	97.77	$4,\!214,\!418$	$17.03$
BiFPN	94.38	${\bf{97}}.{\bf{59}}$	${\bf{98}}.{\bf{26}}$	$1,\!897,\!362$	$15.12$

(c) Performance of lightweight module

In order to balance the detection speed and accuracy of the algorithm, the lightweight ghost module is used to reconstruct the object recognition network. Table 3 verifies the detection performance after using the ghost module, namely, the ghost module is used to replace standard convolution based on experiment (b). For standard convolution, it can be clearly seen from the last two lines of Table 3 that after using the ghost module, the parameters of the model is reduced to 50.4% of original, the amount of computation is reduced to 51.15% of its original magnitude and the detection time of each image is reduced by 6.49 ms, and the detection accuracy is only reduced by 0.2%. It can greatly reduce the parameters and computation of the model by using of ghost module, and has a little effect on the detection accuracy. Compared with the original Yolov4-tiny, the parameters and computations become 66.7% and 64.02% of the original Yolov4-tiny, respectively, and the detection accuracy on the airplane dataset is improved by 7.76%.

**Table 3:** Performance comparison of lightweight modules. The best and the second results are highlighted in bold and underline, respectively.

Model	AP (%)	Params $\downarrow$	Flops (G) $\downarrow$	t (ms) $\downarrow$
Yolov4-tiny	$90.30$	5,874,116	5.17	9.05
(b)-BiFPN	${\bf{98}}.{\bf{26}}$	$7,\!771,\!787$	$6.47$	$15.12$
(b)-BiFPN+ghost	98.06	${\bf{3,\!918,\!603}}$	${\bf{3}}.{\bf{31}}$	${\bf{8}}.{\bf{63}}$

(d) Comprehensive experiment (comparison with other algorithms)

In order to verify the effectiveness of the three improved methods proposed in this article, ablation experiments were added, as shown in Table 4. Experiment A, Experiment B, and Experiment C are the results of gradually increasing the three improved strategies. From the table, it can be seen that each improved method has a relatively obvious effect on Yolov4-tiny. The final model recall, accuracy, and average detection accuracy are 9.06%, 0.33%, and 8.03% higher than Yolov4-tiny, respectively, And the time to detect each image is also 0.42 ms shorter than Yolov4-tiny, which is undoubtedly a lightweight and accurate aircraft detection model for remote sensing images. It further illustrates the effectiveness of the three methods proposed in this article. The use of multi-dimensional attention mechanism and bidirectional weighted feature fusion network can improve the detection accuracy and robustness of the algorithm, and the use of ghost convolution can reduce the amount of calculation and parameters of the model, and improve the detection speed. It shows that the AE-YOLO proposed in this article is a lightweight and efficient aircraft detection model for remote sensing images.

**Table 4:** Ablation experiment.

Groups	LCBAM	BiFPN	Ghost	R/%	P/%	AP/%	t/ms
Yolov4-tiny	$\times$	$\times$	$\times$	85.45	96.96	90.3	9.05
Experiment A	$\surd$	$\times$	$\times$	93.86	95.14	96.19	10.53
Experiment B	$\surd$	$\surd$	$\times$	94.38	97.59	98.26	15.12
Experiment C	$\surd$	$\surd$	$\surd$	94.51	97.29	98.06	8.63

**Figure 10:**
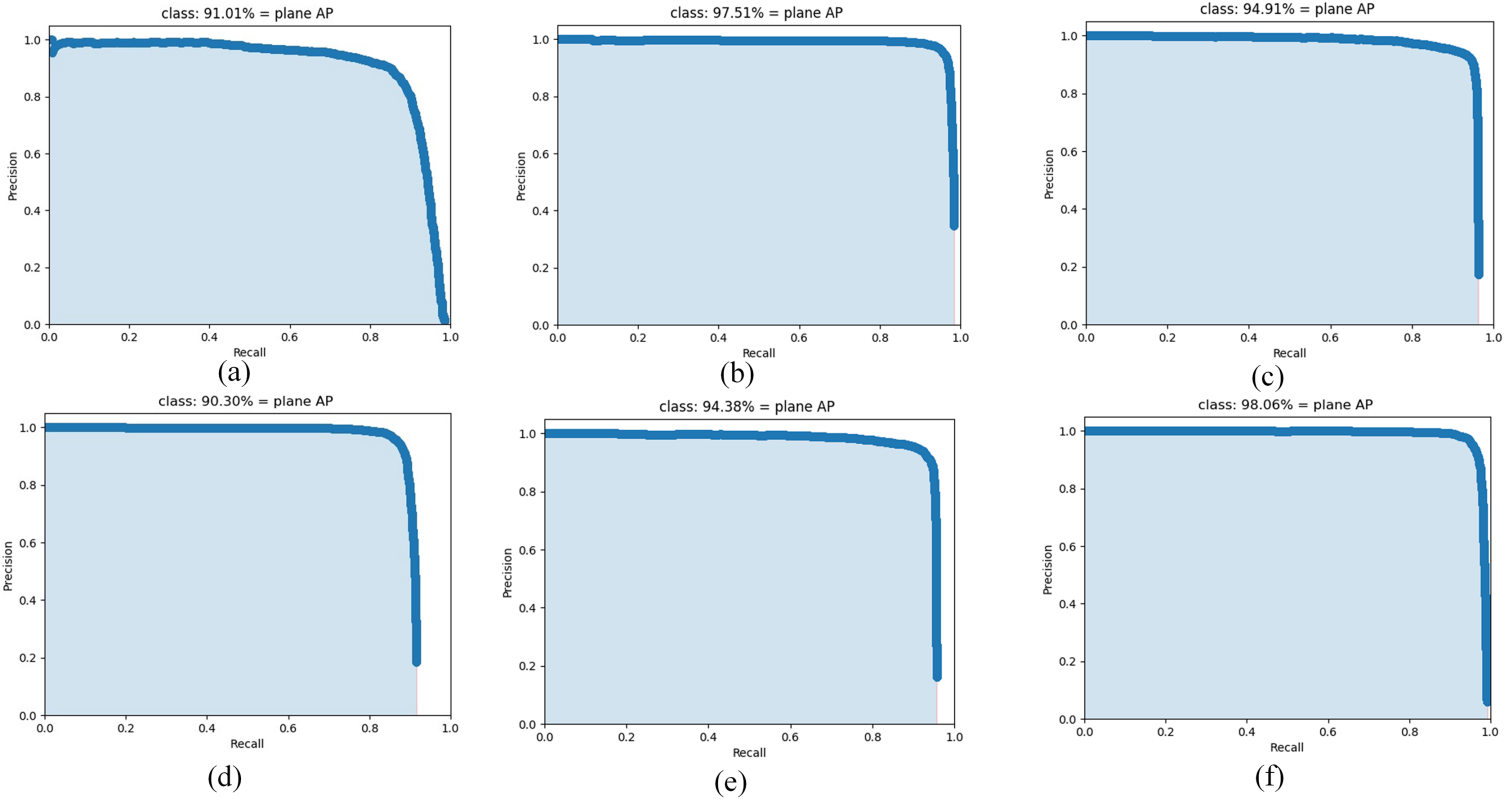
AP comparison of the different algorithms: (A) Yolov3, (B) Yolov4, (C) Vgg-SSD, (D) Yolov4-tiny, (E) Mobilenet-SSD, and (F) AE-YOLO.

In order to further evaluate the performance of the algorithm, this article compares the performance of AE-YOLO algorithm with the state-of-the-art neural network algorithms on the airplane dataset, including large convolutional neural networks Yolov3, Yolov4, Vgg-SSD and lightweight convolutional neural networks Mobilenet-SSD, Yolov4-tiny. The comparison results are listed in Table 5. As shown in Table 5, whether it is a large network or a lightweight network, the average detection accuracy of the algorithm proposed in this article can achieve the best results. Compared with Yolov3, Yolov4, and Vgg-SSD, it has increased by 7.05%, 0.55%, and 3.15%, respectively, and it has increased by 7.76%, 3.68% and 6.93%, respectively, compared with the lightweight Yolov4-tiny and Mobilenet-SSD and Mobilenet-Yolov4. Although the amount of parameters and computation does not reach the minimum value, it can also meet the conditions of deployment, and meanwhile meet the demand for rapid detection of airplane. All of this explained that the three methods proposed in this article are effective. The use of multi-dimensional attention mechanism and bidirectional weighted feature fusion network can improve the detection accuracy and robustness of the algorithm, and the use of ghost convolution can reduce the computation and parameters of the model, and it can improve the detection speed, indicating that the AE-YOLO proposed in this article is a lightweight and efficient airplane detection algorithm for remote sensing images. Figure 10 shows the AP comparison of the different algorithms. As Fig. 10 shows, the AE-YOLO algorithm has the largest area under the PR curve and the best detection performance.

**Table 5:** Performance comparison between different algorithms. The best and the second results are highlighted in bold and underline, respectively.

Model	R (%)	P (%)	AP (%)	Params $\downarrow$	Flops (G) $\downarrow$	t (ms) $\downarrow$
Yolov3	–	–	$91.01$	$61,\!529,\!119$	$49.63$	$37.78$
Yolov4	${\bf{95}}.{\bf{02}}$	$96.51$	97.51	$63,\!937,\!686$	$45.26$	$50.50$
Vgg-SSD	$78.75$	${\bf{97}}.{\bf{65}}$	$94.91$	$23,\!745,\!908$	$87.73$	$23.80$
Yolov4-tiny	$85.45$	$96.96$	$90.30$	$5,\!874,\!116$	$5.17$	$9.05$
Mobilenet-SSD	$81.08$	97.34	$94.38$	${\bf{3,\!675,\!256}}$	${\bf{1}}.{\bf{91}}$	${\bf{8}}.{\bf{54}}$
Mobilenet-Yolov4	$94.51$	$95.30$	$97.23$	$12,\!266,\!614$	$15.26$	–
AE-YOLO	94.51	$97.29$	${\bf{98}}.{\bf{06}}$	3,918,603	3.31	8.63

## Conclusion

Small airplane objects are easily affected by natural environment such as illumination and ground background, there is high missed detection rate and low detection accuracy of small airplane objects in remote sensing images. This article proposes an accurate and efficient algorithm AE-YOLO to the above problems. A lightweight multi-dimensional attention mechanism LCBAM was firstly integrated into the backbone network to enhance the feature extraction capability of the backbone network. Then, a weighted bidirectional feature fusion network was used to fuse the features by making full use of the details of the shallow features and the deep features, and to learn the importance of each branch through self-learning weights to enhance the utilization of features. To balance detection rate and detection accuracy, a lightweight ghost module was used to reconstruct the network in the end. The experimental results show that the proposed AE-YOLO algorithm improves the detection accuracy by 7.76% compared with Yolov4-tiny, and reduces the parameters and computation to 50.4% and 51.15% of the original, respectively. It also has a good detection effect in airplane images with densely distributed in the natural environment, overexposure, and ground looks similar to background in color. The experiments also found that the proposed AE-YOLO algorithm is insufficient for the detection of some objects with serious occlusion. For future work, we will continue to enhance the proposed algorithm and improve its detection performance on the data enhancement of small objects and the detection of objects with serious occlusion and blurred background.

## Additional Information and Declarations

### Data Availability

The following information was supplied regarding data availability: The code is available at GitHub and Zenodo: https://github.com/Lbx2020/AE-Yolo; Lbx2020. (2022). Lbx2020/AE-Yolo: First release of AE-Yolo (v1.0.0). Zenodo. https://doi.org/10.5281/zenodo.7423854. The data is available at: -RSOD dataset: https://github.com/RSIA-LIESMARS-WHU/RSOD-Dataset-#rsod-dataset. -UCAS-AOD dataset: https://github.com/Lbx2020/UCAS-AOD-dataset.
